# A noise-robust deep clustering of biomolecular ions improves interpretability of mass spectrometric images

**DOI:** 10.1093/bioinformatics/btad067

**Published:** 2023-02-06

**Authors:** Dan Guo, Melanie Christine Föll, Kylie Ariel Bemis, Olga Vitek

**Affiliations:** Khoury College of Computer Sciences, Northeastern University, Boston, MA 02115, USA; Khoury College of Computer Sciences, Northeastern University, Boston, MA 02115, USA; Institute for Surgical Pathology, Medical Center – University of Freiburg, Freiburg 79106, Germany; Faculty of Medicine, University of Freiburg, Freiburg 79110, Germany; Khoury College of Computer Sciences, Northeastern University, Boston, MA 02115, USA; Khoury College of Computer Sciences, Northeastern University, Boston, MA 02115, USA

## Abstract

**Motivation:**

Mass Spectrometry Imaging (MSI) analyzes complex biological samples such as tissues. It simultaneously characterizes the ions present in the tissue in the form of mass spectra, and the spatial distribution of the ions across the tissue in the form of ion images. Unsupervised clustering of ion images facilitates the interpretation in the spectral domain, by identifying groups of ions with similar spatial distributions. Unfortunately, many current methods for clustering ion images ignore the spatial features of the images, and are therefore unable to learn these features for clustering purposes. Alternative methods extract spatial features using deep neural networks pre-trained on natural image tasks; however, this is often inadequate since ion images are substantially noisier than natural images.

**Results:**

We contribute a deep clustering approach for ion images that accounts for both spatial contextual features and noise. In evaluations on a simulated dataset and on four experimental datasets of different tissue types, the proposed method grouped ions from the same source into a same cluster more frequently than existing methods. We further demonstrated that using ion image clustering as a pre-processing step facilitated the interpretation of a subsequent spatial segmentation as compared to using either all the ions or one ion at a time. As a result, the proposed approach facilitated the interpretability of MSI data in both the spectral domain and the spatial domain.

**Availabilityand implementation:**

The data and code are available at https://github.com/DanGuo1223/mzClustering.

**Supplementary information:**

Supplementary data are available at *Bioinformatics* online.

## 1 Introduction

**Fig. 1.**
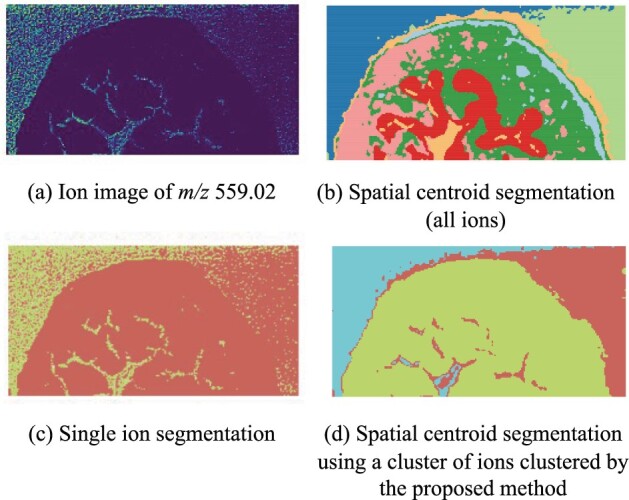
Spatial segmentation taking as input a cluster of ions improves the accuracy of detecting image background. (**a**) Ion image of *m*/*z* 559.02. (**b**) Multivariate spatial shrunken centroid segmentation (Bemis *et al.*, 2016) based on all the 746 ions. (**c**) Univariate segmentation using spatial-Dirichlet Gaussian Mixture Model (Guo *et al.*, 2019a) of ion m/z=559.02. (**d**) Spatial centroid segmentation based on a cluster of ions generated by the proposed approach. The matrix area inside the urothelium is shown in cyan (b), green-yellow (c) and cyan and red (d)

Mass Spectrometry Imaging (MSI) characterizes the spatial distribution of metabolites, lipids, proteins and other biomolecules in complex samples such as tissues (Buchberger *et al.*, 2018). It has been successfully used for mapping protein distribution of intact tissues (Balluff *et al.*, 2011; Liu *et al.*, 2021), for discriminating tumor versus healthy tissues (Calligaris *et al.*, 2015; Föll *et al.*, 2022; Guo *et al.*, 2020) and for the discovery of protein biomarkers of disease (Schwamborn, 2012). MSI experiments collect mass spectra at different locations on a sample (often referred to as *pixels*) in a raster pattern with spatial resolution from 1 to 150 μm. One interpretation of MSI experiments focuses on the resulting mass spectra. A mass spectrum is viewed as a graph with mass over charge (*m*/*z*) ratios of the ionized molecules on the *x*-axis and the intensities relating to the ion abundances on the *y*-axis. The interpretation of mass spectrum mainly includes *m*/*z* identification and *m*/*z* relations, i.e. whether they are likely from a same source based on the spatial distribution of *m*/*z*. Another interpretation of MSI experiments depicts intensities of one *m*/*z* or of aggregates of several *m*/*z*s across all the pixels. Such image shows the spatial distribution of the ions in the tissue, and is referred to as *ion image*. Figure 1a shows an example ion image of an MSI experiment for mouse bladder tissue (described in more detail in Section 4.2).

Although MSI contains the word ‘imaging’ in its name, its data are very different from data produced by other bioimaging techniques. Unlike in natural images, where a pixel is represented by a small number of channels, MSI data are complex and high dimensional in both spectral and spatial domains. In the spectral domain, each spectrum contains tens of thousands of *m*/*z* features. Multiple *m*/*z* may have similar spatial distributions. The similarity may appear for technological reasons, for example when the *m/z* are derived from a same molecule by fragmentation, forming multiple isotope variants or forming sodium or potassium adducts (Guo *et al.*, 2020; Janda *et al.*, 2021). Multiple *m*/*z* may also have similar spatial distributions for biological reasons, e.g. if they are derived from molecules specific to a tissue compartment, cell type or biomolecular pathway. In the spatial domain, an MSI image consists of tens of thousands of spectra, with patterns reflecting the morphology of tissues such as anatomic structures or histology types.

The high dimensionality of MSI data has motivated the development of unsupervised clustering methods in the spectral domain. These methods group *m*/*z* into clusters according to the similarity of their spatial distributions (Alexandrov *et al.*, 2013; Wüllems *et al.*, 2019; Zhang *et al.*, 2021). The resulting clusters have numerous applications ranging from technological quality control and background detection to improved analyte identification and biological process characterization (Janda *et al.*, 2021; Ovchinnikova *et al.*, 2020).

Some frequently used ion clustering methods summarize the ion image in a single vector of *m*/*z* (Alexandrov *et al.*, 2013; Wüllems *et al.*, 2019). Unfortunately, these methods lose the 2D information in the images, and as a result, are not able to learn the spatial contextual features. Alternatively, recent advances in Deep Neural Networks (DNNs) inspired researchers to first use DNN trained on natural image classification tasks as feature extractors, and then apply clustering on the extracted features instead of the original ion images (Zhang *et al.*, 2021). However, when a DNN is trained on natural images as opposed to ion images, the extracted features may be undermined by the mass spectrometric noise and not well represent the spatial distribution of ions.

This manuscript contributes a novel approach for unsupervised clustering in the spectral domain. Similar to the most recent work (Zhang *et al.*, 2021), we rely on DNN to learn spatial features. Unlike the existing methods, the proposed approach does not train DNN on natural images for feature extraction. Instead, it trains a Convolutional Neural Network (CNN) on ion images directly, to simultaneously extract high-level spatial features, and learn cluster labels. The proposed approach adopts a convolutional autoencoder to denoise the ion images prior to the clustering CNN, making feature learning more robust to noise. This architecture does not require us to separate feature learning from clustering, thus simplifying the training process. Finally, although the overall ion clustering task is unsupervised, the network is trained in a ‘supervised’ manner with a pairwise classification loss and adaptive self-paced training.

We evaluated the utility of the proposed approach on a simulated dataset with known ground truth, and on four experimental datasets of different tissue and ionization types. The evaluation was performed from two perspectives. The first perspective focused on the interpretation of the spectral domain, specifically whether the proposed approach generated meaningful *m*/*z* clusters. In all the evaluations, the proposed approach clustered ions from a same source more frequently than with the currently available methods. The second evaluation perspective focused on the downstream interpretation of the data in the spatial domain. We showed that considering ions in the representative cluster profiles, and using them as input to existing methods of spatial segmentation could improve the interpretability of spatial segmentation. On the one hand, it could overcome the interpretability limitations of multivariate segmentation as the cluster profile can better isolate subtle signals from rare sources. On the other hand, it is more robust to the noise, thus overcoming the limitations of single ion segmentation by being more robust to noise. For example, segmentation in Figure 1d was less noisy and provided overall more specific insight as compared to both (b) and (c). The proposed approach is implemented in PyTorch and Python.

## 2 Background

In this section, we introduce the background of MSI data preprocessing (Supplementary Section S1.1), spectral domain clustering (Supplementary Section S1.2), spatial domain segmentation and image clustering in computer vision.

### 2.1 Unsupervised segmentation of the spatial domain

In contrast with spectral domain clustering, spatial domain segmentation segments the pixels according to the similarity of mass spectra (Abdelmoula *et al.*, 2021; Alexandrov *et al.*, 2010; Bemis *et al.*, 2016; Guo *et al.*, 2019a; Hu *et al.*, 2021). The resulting segments are regions of the tissue with homogeneous chemical composition. They are useful for virtual micro-dissection of different tissue and cell types, unraveling hidden molecular patterns such as intratumor heterogeneity (Vaysse *et al.*, 2017), or simply for technical reasons such as detecting image background. The methods are either multivariate (Abdelmoula *et al.*, 2021; Alexandrov *et al.*, 2010; Bemis *et al.*, 2016; Hu *et al.*, 2021), i.e. they simultaneously consider all the *m*/*z* of the spectra for pixel segmentation, or univariate (Guo *et al.*, 2019a), i.e. they segment pixels of an ion image for one *m*/*z* at a time. Some multivariate algorithms for unsupervised segmentation of the spatial domain, such as spatial K-means segmentation (Alexandrov *et al.*, 2010) and Spatial Shrunken Centroid (SSC) segmentation (Bemis *et al.*, 2016), delineate regions of homogeneous chemical composition by clustering pixels with similar multivariate spectra. The SSC segmentation method accounts for the spatial correlations between pixels and uses a shrinkage parameter to select important ions, thus providing less noisy spatial segments. An alternative multivariate approach first reduces the dimensionality of the mass spectra, e.g. with Principle Component Analysis (PCA) (Race *et al.*, 2013) or t-Distributed Stochastic Neighboring Embedding (t-SNE) (Abdelmoula *et al.*, 2016), maps them into 1D, and then plots the scores of the mapping on each pixel of the tissue. Pixels with similar scores are then interpreted as regions of similar chemical composition. More recent methods segment other types of lower-dimensional embeddings. For example Abdelmoula et al. (2021) embedded the mass spectra with a Variational Autoencoder (VAE), and subsequently segmented the embedding with a Gaussian Mixture Model (GMM). Multivariate segmentation suffers from the curse of dimensionality, where signals with higher intensity and higher variation dominate other subtle but important signals. For example, multivariate segmentation of the mouse bladder tissue in Figure 1b fails to accurately segment the matrix near the urothelium (segment in orange).

In contrast, univariate segmentation segments pixels for one *m*/*z* at a time. Thresholding methods segment a single ion image by applying one or several intensity thresholds (Hu *et al.*, 2021). Spatial-Dirichlet Gaussian Mixture model (spatial-DGMM) (Guo *et al.*, 2019a) models the intensities of pixels as sampled from spatially dependent Gaussian components, and segments the pixels according to their Gaussian component membership. Univariate segmentation is undermined by random variation in spectral intensities, and produces noisy segments. For example, the univariate segmentation in Figure 1c captures more spatial details, but has less accurate segmentation since one *m*/*z* only carries a limited amount of information.

In this manuscript, we will use the interpretability of the spatial segmentation with method Spatial Shrunken Centroids (SSC) as a criterion for evaluating the usefullness of *m/z* clustering using the subsets of ions.

### 2.2 Unsupervised image clustering in computer vision

Image clustering is of course an important problem in computer vision, with many existing solutions. Deep clustering, i.e. clustering with DNNs, has out-performed conventional non-deep learning methods on most benchmarks (Chang *et al.*, 2017; Rebuffi *et al.*, 2021; Xie *et al.*, 2016). In particular, CNNs or autoencoders automatically learn the visual properties of an image or of an object (Weinmann, 2013). Some popular CNN models include ResNet (He *et al.*, 2016), VGG (Simonyan and Zisserman, 2014) and Xception (Chollet, 2017). Unfortunately, noise in the input images can lead to a significant decrease in CNN performance, possibly due to the removal of some necessary texture features (Karahan *et al.*, 2016; Lau *et al.*, 2021).

Some deep clustering approaches construct a clustering loss, such as K-means or GMM clustering loss, and optimize the loss in the space of features learned by neural networks such as autoencoders (Guo *et al.*, 2017; Xie *et al.*, 2016) or generative adversary networks (GANs) (Mukherjee *et al.*, 2019; Yu and Zhou, 2018). Alternatively, to benefit the feature learning in a ‘supervised’ manner, some approaches such as Deep Adaptive Clustering (DAC) (Chang *et al.*, 2017) train the network with a binary classification loss instead of a clustering loss. The binary classification loss relies on pairwise pseudo-labels between images that indicate whether two images belong to a same cluster (Chang *et al.*, 2017; Guo *et al.*, 2019b; Rebuffi *et al.*, 2021). The term ‘pseudo’ refers to the fact that the pairwise labels used in the loss are not provided by a ground-truth, but are derived from binary pairwise similarities between the images. The pairwise similarities can be quantified using multiple metrics, such as Euclidean, cosine or kernel-based.

Self-paced learning of unsupervised networks (Chang *et al.*, 2017; Guo *et al.*, 2019b; Kumar *et al.*, 2010; Rebuffi *et al.*, 2021) trains the networks in ways that mimic human learning. Self-paced learning starts by learning coarse clusters using samples with high-confidence cluster membership (i.e. highly similar and highly dissimilar image pairs). It then proceeds by gradually learning from images with ambiguous cluster membership. This process is more effective than training on all samples at once since it is able to capture discriminate features rapidly in early training phases.

Unfortunately, none of the methods above are directly applicable to MSI. Due to large biological and technical variation between pixels, and due to the subtle nature of many biomolecular signals, mass spectrometry images tend to be a lot noisier than natural images. We borrow the idea of pseudo-labeling and self-paced training from Chang *et al.* (2017), Rebuffi *et al.* (2021) and Guo *et al.* (2019b), and develop a deep clustering method for ion image clustering that is robust to noisy ion image data.

## 3 Materials and methods

**Fig. 2.**
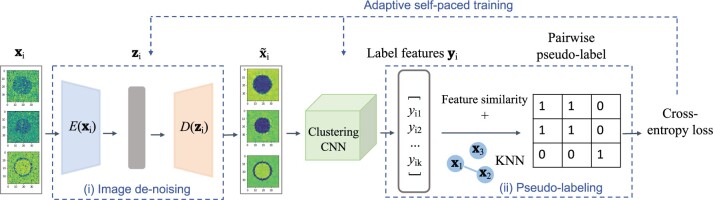
Overview of the proposed noise-robust deep clustering model. The network contains two components: (i) image denoising and (ii) pseudo-labeling. A convolutional autoencoder in component (i) denoises the ion images, and the denoised images are fed to a clustering CNN. Component (ii) assigns pseudo-labels of pairs of images, indicating whether the pair belongs to a same cluster. Model weights are learned iteratively with adaptive self-paced training

The proposed approach for clustering *m*/*z* is summarized in Figure 2. First, we introduce the architecture of the network that mainly contains two components: (i) image denoising and (ii) pairwise pseudo-labeling. Then we introduce adaptive self-paced training to train the parameters of the network containing these two components.

The purpose of component (i) is to account for variations in ion intensities across the pixels from a spatial segment, and generate ‘clean’ images for clustering. Similarly to DAC (Chang *et al.*, 2017) in computer vision, component (ii) learns the visual features of the ion images with a CNN, evaluates similarities between all pairs of the features, and pseudo-labels the image pairs according to whether they belong to a same cluster. To stabilize the process, the pseudo-labels must satisfy the constraint, which states that the nearest neighbors of an ion image should belong to the same cluster as the image. The pseudo-labels are then used to construct the loss function. The network parameters are trained with adaptive self-paced training by gradually including ion images from the training set. After the training, the clustering labels are directly derived from the output of the clustering CNN in component (ii). Finally, all the ions of a cluster are used as input for the downstream multivariate spatial segmentation.

### 3.1 Notation

Let $x_{i}$ represent an ion image *i*, i.e. a 2D array of dimensions *w *×* h*, where *w* and *h* are the width and height of the ion image, and values are ion intensities at each spatial location. Let $X={\left\{x_{i}\right\}}_{i=1}^{n}$ denote the collection of *n* ion images corresponding to *n m*/*z*. We denote $z_{i}$ a *d*-dimensional embedding vector of $x_{i}$, and denote ${\tilde{x}}_{i}$ the image of $x_{i}$ reconstructed by the autoencoder. ${\tilde{x}}_{i}$ is of the same dimensions as $x_{i}$. Finally, $y_{i}=[y_{i1},y_{i2},\ldots ,y_{ik}]$, a vector of the size of the pre-specified number of clusters *k*, is the output of CNN corresponding to ${\tilde{x}}_{i}$.

### 3.2 Image denoising

Prior to learning the visual features, ion images are denoised with a convolutional autoencoder. Let E(·) denote the encoder, and D(·) denote the decoder. The encoder E(·) consists of convolutional layers and decoder D(·) consists of transposed convolutional layers. The reconstructed image ${\tilde{x}}_{i}$ can be written as ${\tilde{x}}_{i}=D(z_{i})=D(E(x_{i}))$. $W_{ae}$ is the weight matrix of *E* and *D*. The loss function of the autoencoder is defined as the Mean Square Error loss between $x_{i}$ and ${\tilde{x}}_{i}$, i.e.

(1) $$L_{ae}(W_{ae})=\sum_{i}||x_{i}-{\tilde{x}}_{i}|{|}^{2}$$

### 3.3 Pairwise pseudo-labeling

Similarly to Chang *et al.* (2017) and Rebuffi *et al.* (2021), we assign to a pair of ion images a pseudo-label that indicates whether they belong to a same cluster. The generation of pairwise labels is similar to that in Chang *et al.* (2017), in that we use the similarity of label features predicted by a CNN. However, unlike Chang *et al.* (2017), we stabilize the training by introducing an additional constraint, which requires that each K-nearest-neighbor of an ion image is in the same cluster as that image.

**Similarity of label features.** Let $y_{i},y_{j}$ denote the cluster label features of ion images $x_{i}$ and $x_{j}$. Let $f(W_{c})$ denote the clustering CNN, and $W_{c}$ the parameters of *f*, such that $y_{i}=f({\tilde{x}}_{i};W_{c})$. The last layer of the clustering CNN is a softmax activation layer, which enforces the non-negativity and the clustering constraints on the feature labels. We define a matrix **S** of dimension *n *×* n* whose elements containing the cosine similarities.

(2) $$s_{ij}(y_{i},y_{j})=\frac{y_{i}\cdot y_{j}}{\left||y_{i}||||y_{j}|\right|}$$

We further assign to a pair of images *i* and *j* a binary label $A_{ij}^{\prime }$, indicating whether the two images belong to a same cluster

(3) $$A_{ij}^{\prime }=\{\begin{array}{cc} 1, & s_{ij}>=ub\\ NA, & lb<s_{ij}<ub\\ 0, & s_{ij}<=lb \end{array}$$

The thresholds *ub* and *lb* (*lb* < *ub*) are respectively the lower bound for the similarity scores in a *same* cluster, and the upper bound for the similarity scores in *different* clusters. Any pair with a similarity above *ub* belongs to a same cluster. Any pair with a similarity below *lb* belongs to a different cluster. To allow dataset-specific flexibility, we initialize these values according to the lb=45th and ub=95th percentiles of the similarity scores of all the pairs of ion images, and adjust them subsequently during adaptive self-paced training.

**K-nearest-neighbors at the level of input images.** Since the clustering CNN is not pre-trained, a random initialization sometimes results in local minima without meaningful clusters. To stabilize the training, in addition to Equation 3, we further assign the cluster membership label at the level of input images. Specifically, if image *i* belongs to the K-nearest-neighbors of image *j* or vice versa, the re-defined binary label *A_ij_* of image pair *i* and *j* is written as:

(4) $$A_{ij}=\{\begin{array}{cc} 1, & x_{j}\in \mathrm{KNN}(x_{i})\;\text{or}\;x_{i}\in \mathrm{KNN}(x_{j})\\ A_{ij}^{\prime }, & \mathrm{otherwise} \end{array}$$

### 3.4 Adaptive self-paced training

Algorithm 1 Adaptive self-paced training

**Input:** $X={\left\{x_{i}\right\}}_{i=1}^{n}$

**Parameters:** *k—*number of clusters

 *η* - learning rate of model weights

 *θ* - stopping threshold

 *ϵ* - learning step of *λ*

 *epochs—*number of pre-training epochs

 *Epochs—*number of training epochs

 *iterations—*number of training iterations

**Output:** cluster labels *c_i_* for i=1,2,…,n

______________________________________________________

1: ▷ *Stage 1 - pre-training component (i)*

2: **for** e from 1 to *epochs* **do**

3: $W_{ae}\leftarrow W_{ae}-\eta \frac{\partial L_{ae}(W_{ae})}{\partial W_{ae}}$

4: **end for**

5: ▷ *Stage 2 - Adaptive self-paced training of components (i) and (ii)*

6: $S\leftarrow \{s_{ij}\},\;i,j\in \{1,2,\ldots ,n\}$ (Equation 2)

7: $lb\leftarrow P_{45}(S),\;ub\leftarrow P_{95}(S),\;\lambda \leftarrow ub-lb$

8: **for** e from 1 to *Epochs* **do**

9:  **for** iter from 1 to *iterations* **do**

10:   ${\left\{y_{i}\right\}}_{i=1}^{n}=f({\left\{{\tilde{x}}_{i}\right\}}_{i=1}^{n};W_{c})$

11:   Compute pseudo-label matrix *A* (Equations 3 and 4)

12:   $W_{ae}\leftarrow W_{ae}-\eta \frac{\partial L(W_{ae},W_{c})}{\partial W_{ae}}$

13:   $W_{c}\leftarrow W_{c}-\eta \frac{\partial L(W_{ae},W_{c})}{\partial W_{c}}$

14:  **end for**

15:  $S\leftarrow \{s_{ij}\},\;i,j\in \{1,2,\ldots ,n\}$ (Equation 2)

16:  $l=l+0.8\cdot \epsilon ,\;u=u-0.2\cdot \epsilon ,\;lb\leftarrow P_{l}(S),\;ub\leftarrow P_{u}(S)$

17:  λ←ub−lb

18: **end for**

19: **return:**

20: $y_{i}=f(D(E(x_{i}));W_{c})$

21: $c_{i}={\text{arg max}}_{p}$*y_ip_* for p={1,2,…,k}

Algorithm 1 describes the training of the network containing the convolutional autoencoder and the clustering CNN. The algorithm proceeds in two stages. The first stage pre-trains the autoencoder to minimize the reconstruction loss of component (i) in Equation 1 (lines 2–4). The second stage trains the clustering CNN in component (ii) and fine-tunes the autoencoder with self-paced training. In the beginning, the network is only trained on some extremely confidently labeled pairs, to learn representative features for coarse clustering. Then it adds less confidently labeled image pairs to fine-tune the clustering. As a result, the process gradually increases the total number of confidently labeled image pairs. We describe the details of this second stage of the training next.

First we obtain the similarity matrix *S* according to Equation 2 (line 6) and initialize *ub* as the 95th percentile of the pairwise similarity values, and *lb* as their 45th percentile (line 7). Once we obtain the pseudo-labels *A_ij_*, the clustering loss is defined as

(5) $$\begin{array}{c} L_{c}(W_{c})=\sum_{i,j}v_{ij}g(A_{ij},s(y_{i},y_{j};W_{c}))\\ s.t.\;lb<ub\\ \text{where}\;v_{ij}=1\;\text{if}\;A_{ij}\in \{0,1\}\;\text{else}\;0 \end{array}$$

The constrain *lb* < *ub* ensures that during the training process, the updated lower bound remains below the updated upper bound. Given the image pair labels, we transform the loss $g(A_{ij},s(y_{i},y_{j};W_{c}))$ into a classification loss. Here we use the cross-entropy loss that is most common for classification problems:

(6) $$\begin{array}{c} g(A_{ij},s(y_{i},y_{j};W_{c}))=\\ -A_{ij}\;\text{log}(s(y_{i},y_{j};W_{c}))-(1-A_{ij})(1-\log(s(y_{i},y_{j};W_{c}))) \end{array}$$

Next, the adaptive self-paced training trains the clustering CNN $f(W_{c})$ and fine-tunes the parameters $W_{ae}$ of the autoencoder (lines 12 to 13) by minimizing

(7) $$\begin{array}{c} \text{min}\;L(W_{ae},W_{c},\lambda )=\sum_{i,j}v_{ij}g(A_{ij},s(y_{i},y_{j}))+\lambda +\sum_{i}||x_{i}-{\tilde{x}}_{i}|{|}^{2}\\ s.t.\;lb<ub\\ \text{where}\;v_{ij}=1\;\text{if}\;A_{ij}\in \{0,1\}\;\text{else}\;0 \end{array}$$

The regularization parameter *λ* in Equation 7 penalizes the number of image pairs not selected to train the neural network. Since *ub* and *lb* control the number of samples to be pseudo-labeled, we simply set *λ* as λ=ub−lb, i.e. the gap between the upper and the lower bounds of the pairwise similarity scores. According to Equation 3, smaller *λ* corresponds to a larger number of selected image pairs, and *λ *= 0 corresponds to all the image pairs being selected.

Since the indicator *v_ij_* is a deterministic function of *A_ij_*, the number of non-zero values of *v_ij_* is also adjusted by the value of *λ*. We set *ub* as the *u_th_* percentile and *lb* the *l_th_* percentile of *S* and we adjust *ub* and *lb* by decreasing *u* with u=u−0.2·ϵ, and increasing *l* with l=l+0.8·ϵ (line 17), where *ϵ* is the learning step. Since λ=ub−lb, the value of *λ* decreases with each training epoch.

Note that we do not have the ground truth of clusters, and the method does not utilize any image labels. We did not split the data into training data and validation data for the purpose of grouping all *m*/*z* in an MSI experiment into clusters.

### 3.5 Assigning ion image clusters and tissue segments

The fully trained neural network outputs non-negative label features $y_{i}$ with *L*_1_ norm of 1. Thus the cluster membership of each ion image is

(8) $$c_{i}={\text{arg max}}_{p}\left\{y_{ip}\right\},\;\text{where}\;p\in \{1,2,\ldots ,k\}$$

### 3.6 Implementation

We implemented the proposed approach using PyTorch. In all the examples in this manuscript, the convolutional autoencoder contained two convolutional layers in the encoder and two transposed convolutional layers in the decoder, and the embedding dimension was 7. The clustering CNN contained seven convolutional layers with batch normalization and ReLU activation, and one fully connected layer with Softmax activation. The experiments were conducted on a hardware with 13 GB RAM, 2 Intel(R) CPUs and 1 NVIDIA Tesla T4 GPU. The training took several minutes on the small simulated dataset and about one hour on the large experimental datasets. For different datasets, the model was trained from a random initialization if not otherwise specified. We did not jointly train the model across different datasets. The spatial domain segmentation was performed using Spatial Shrunken Centroids (SSC) in the open-source *Cardinal* package (Bemis *et al.*, 2015), for reasons of performance and easy-to-use implementation.

## 4 Evaluation strategy of clustering

### 4.1 Methods used for the evaluation

We compared the performance of the proposed approach to methods that vectorize ion images, i.e. K-means and GMM. We further compared the proposed approach to representation-based methods that account for the spatial features of the images, i.e. variational autoencoder (VAE) and Xception followed by K-means. The details of the implementation are described in Supplementary Section S3.1. All the ion images were normalized to [0,1] prior to clustering.

### 4.2 Evaluation on a simulated dataset

Interpreting ion image clusters is challenging due to the lack of ground truth of cluster labels. Therefore, we first evaluated the proposed approach on a simulated dataset, where the ground truth is known.

**Fig. 3.**
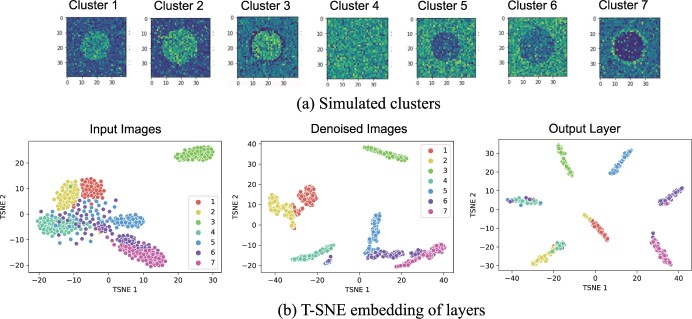
On the simulated dataset, the t-SNE embedding shows that the denoised images generated by the autoencoder and the output layer of the clustering CNN distinguish different clusters. (**a**) Representative ion images in each simulated cluster. (**b**) The t-SNE embedding plot of the input images, of the denoised images $\tilde{x}$ and of the CNN output **y**. Colors indicate the true cluster labels

**Simulated dataset.** We simulated seven clusters of ion images of dimension 40 × 40, with 100 *m*/*z* features in each cluster. The spatial distribution of representative ion images for each cluster is shown in Figure 3a. The ion images have two to three segments (clusters 1–3, 5–7) or are homogeneously distributed across the whole sample (cluster 4). It is often the case in MSI that one ion produces an opposite image of another. Clusters 1–3 mimic this scenario by having opposite images of clusters 5–7. The details of simulation can be found in Supplementary Section S2.1.

**Evaluation on the simulated dataset in the spectral domain.** Since the simulated dataset had known ground truth of cluster membership, we used common evaluation metrics, including clustering accuracy (ACC), Normalized Mutual Information (NMI) and Adjusted Rand Index (ARI). The metrics range between 0 and 1, with higher values indicating better clustering. The definition of NMI and ARI can be found in Chang *et al.* (2017). We explain the calculation of ACC in detail in Supplementary Section S3.2.

### 4.3 Evaluation on experimental datasets

To evaluate the ability of the proposed approach to facilitate the interpretation of MSI experiments, we considered four additional public datasets acquired on different tissue types with different ionization sources. The mouse urinary bladder dataset (Römpp *et al.*, 2010) is a benchmark dataset downloaded from the public repository PRIDE (PXD001283). The mouse kidney (ID: 2019-07-19_19h41m43s), mouse brain (ID: 2021-03-22_15h30m15s) and lymph node datasets (ID: 2021-03-30_17h37m20s) were downloaded from *METASPACE*. The datasets were preprocessed with the *Cardinal* package in *R* with standard steps, including total ion current (TIC) normalization, peak detection, peak filtering and peak binning. The signal-to-noise was set to 3. The binning resolution was set to 200 ppm. The detailed description of the datasets can be found in Supplementary Section S2.2.

**Evaluation on the experimental datasets in the spectral domain.** We evaluated the proposed approach on the experimental datasets from two perspectives. The first perspective is the interpretation of *m*/*z* assigned to a same cluster. The datasets in this manuscript lacked tandem mass spectra, making it difficult to identify the underlying analytes. As a result, it was not possible for us to evaluate the biological plausibility of the ion image clusters, such as whether proteins in a same cluster belong to a same protein complex. Despite this limitation, the high mass resolution of these experiments allowed us to identify multiple isotopes of a same ion, and examine whether these isotopic ion images were assigned to a same cluster. The strategy we used to identify isotopic ions was as follows. Let Δm denote the mass difference between two *m*/*z* features and *p* denote the Pearson Correlation between their ion images. If |Δm−1.003|<δ and *p *>* *0.7, the two *m*/*z* were considered as isotopic ions. We set *δ* as 0.01 for all the experimental datasets.

Similarly to Zhang *et al.* (2021), we used isotope ratio and relative isotope ratio as the evaluation metrics. Isotope ratio is defined as the fraction of the number of isotopic pairs predicted in the same cluster among the total number of isotopic pairs. A higher ratio indicates better clustering performance. In a perfect clustering, the isotope ratio equals 1. Unfortunately, the isotope ratio above favors large clusters. For example, a maximal cluster containing all the ion images produces an isotope ratio close to 1, but it is a suboptimal cluster. The relative isotope ratio is an alternative metric that accounts for this artifact. It is defined as the isotope ratio of the predicted clusters divided by the isotope ratio of a random clustering with clusters of the same size.

**Evaluation on the experimental datasets in the spatial domain.** The second perspective for evaluating the outcome of ion image clustering is the downstream interpretation of the data in the spatial domain. We qualitatively evaluated the spatial segments by relating the segments to anatomic structures. Specifically, the evaluation considers the ions in the representative cluster profiles, and uses them as input to the Spatial Shrunken Centroid (SSC) segmentation method. We used the mean ion image that depicts the average intensity of ions across pixels as a representation of the overall spatial pattern of ions in a cluster. There was no feature selection or aggregation performed prior to the segmentation, and the shrinkage parameter of SSC segmentation was set as 0. We qualitatively compared the spatial segments using the ions in a cluster and using all ions. The segmentation was considered more interpretable if it generated more detailed and less noisy segments related to anatomic structures.

## 5 Results

### 5.1 On the simulated dataset, the proposed approach achieved the highest clustering accuracy

The t-SNE embedding of the simulated ion images in Figure 3b illustrates the difficulty of the image clustering task in the simulated dataset. Due to the relatively large noise, only cluster 3 was easy to distinguish from the other clusters. The remaining clusters, in particular clusters 5–7, were mixed together. The denoised images were better separated than the original ion images, especially in clusters 4–7. The clusters were further separated by the output layer of the neural network.

Table 1 summarizes the clustering performance of K-means, GMM, autoencoder and the proposed approach with the correctly specified number of clusters *k *=* *7. Vectorization-based K-means and GMM were sensitive to noise, especially in the high-dimensional feature space, and had low clustering accuracy. Representation-based VAE captured the spatial contextual features of images, and substantially outperformed K-means and GMM. However, the clustering based on the features extracted by Xception trained on ImageNet showed a low clustering accuracy on the simulated dataset. This may be due to the difference of representative features between natural images and the simulated images in this dataset. Overall, the proposed approach achieved the best clustering performance in terms of all the evaluation metrics. Therefore the proposed approach enabled a more accurate interpretation of ions from a same source.

**Table 1.** On the simulated dataset, the proposed approach achieved the highest ACC, NMI and ARI as compared to K-means, Gaussian Mixture Model (GMM), Variational Autoencoder (VAE) and Xception

	K-means*	GMM*	VAE	Xception	Proposed
ACC	0.65	0.75	0.89	0.58	0.95
NMI	0.72	0.72	0.80	0.56	0.89
ARI	0.57	0.60	0.77	0.39	0.85

*Note*: Starred methods vectorize the ion images.

### 5.2 On the experimental data, the proposed approach achieved the highest isotope ratios of clustered ions

We specified 6 clusters in the mouse bladder dataset, 10 clusters in the mouse kidney dataset, 6 clusters in the mouse brain dataset and 8 clusters in the human lymph node dataset. The number of clusters was selected by manually inspecting the t-SNE embedding of all the ion images. Table 2 compares the performance of K-means, GMM, VAE, Xception and the proposed approach with respect to two strategies of identifying isotope ions. Except for the relative isotope ratio of the brain dataset in Table 2, the proposed approach achieved the best isotope ratio and best relative isotope ratio for all four datasets.

**Table 2.** On the experimental datasets, the proposed approach achieved higher isotope ratio and relative isotope ratio compared to K-means, Gaussian Mixture Model (GMM), Variational Autoencoder (VAE) and Xception

	K-Means*		GMM*		VAE		Xception		Proposed	
	IR	RIR	IR	RIR	IR	RIR	IR	RIR	IR	RIR
Bladder	0.88	1.40	0.88	1.95	0.88	3.75	0.90	4.38	0.96	4.80
Kidney	0.79	4.29	0.82	5.06	0.82	6.93	0.75	6.82	0.90	8.67
Brain	1.00	5.81	1.00	6.49	1.00	7.19	1.00	6.80	1.00	6.51
Lymph node	0.90	4.68	0.92	4.79	0.81	3.91	0.90	4.12	0.95	4.83

*Note*: Starred methods vectorize the ion images.

IR, isotope ratio; RIR, relative isotope ratio.

### 5.3 The proposed approach produced more homogeneous clusters

**Fig. 4.**
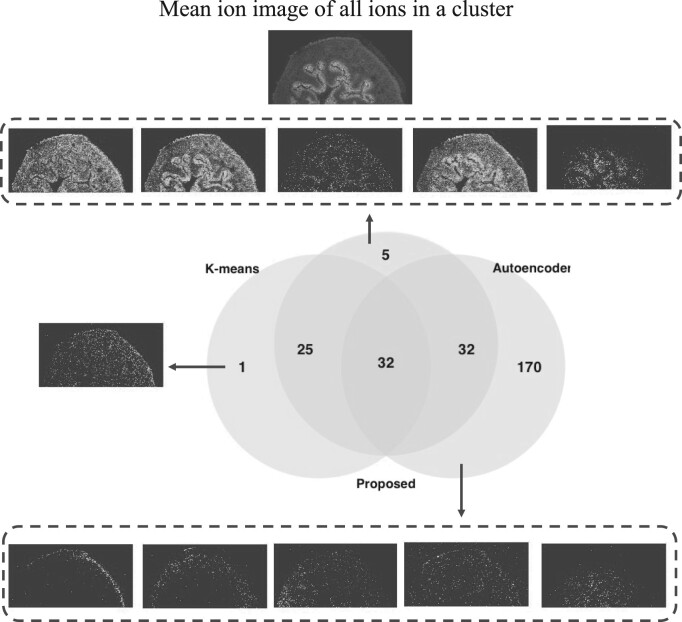
On the mouse urinary bladder dataset, the proposed approach clustered *m*/*z* images with more similarity to the cluster mean ion image. The numbers in the Venn diagram are the number of *m*/*z* images in the reference cluster. The plot characterizes the intersection of *m*/*z* images in clusters, which were generated by K-means, VAE and the proposed approach, and had a similar mean ion image. The figure highlights ion images of *m*/*z* uniquely included by each method

Figure 4 characterizes the *m*/*z* images of the mouse urinary bladder dataset clustered together by all the methods. These ion images had a similar mean ion image. The figure also shows *m*/*z* that were uniquely added to the cluster by only one of the methods. The one *m*/*z* uniquely included by K-means had homogeneous distributions over the tissue area. VAE included 170 more *m*/*z* than either K-means or the proposed approach. However, the spatial distribution of these *m*/*z* did not resemble the mean ion image. Figure 4 shows five ion images randomly selected from these 170 *m*/*z*. The images presented no clear morphology in the urothelium area of the bladder. In contrast, most of the *m*/*z* uniquely included by the proposed approach had higher intensities in the urothelium area, as shown in Figure 4. These images are consistent with the spatial distribution pattern of the mean ion image. Overall, the proposed approach produced clusters in which ions have more similar distributions and more accurately interpreted *m*/*z* that are likely from a same source. In addition, the ion cluster profiles can be exploited in feature selection and it is more effective than intensity-based feature selection strategy (Supplementary Section S4.2).

### 5.4 Ablation study positively associated the autoencoder and the KNN with accuracy of ion images clusters

In order to characterize the impact of each component of the proposed approach on its overall performance, we conducted an ablation study considering three aspects: (i) the autoencoder for image denoising, (ii) the use of KNN in pairwise pseudo-labeling and (iii) ResNet-18 (He *et al.*, 2016) pre-trained on the ImageNet. The intention behind the pre-trained ResNet-18 was to replace the convolutional encoder and the clustering CNN using ResNet-18 pre-trained on the ImageNet. We aimed to investigate whether a pre-trained complicated network can learn spatial patterns of ion images better than a relatively lightweight CNN without pre-training on other sources of images. This can hint at the impact of large model size and prior knowledge of natural images. We chose ResNet-18 (with 11 M parameters) as an example, within the limit of our computational resources. As shown in Table 3, denoising autoencoder was associated with a substantial performance gain in terms of both the isotope ratio and the relative isotope ratio criteria. Pre-trained ResNet-18 did not result in a significant improvement. This may be due to the fact that ion images are very different from natural images.

**Table 3.** On the mouse kidney dataset, the autoencoder, the use of KNN in the pseudo-labeling was positively associated with the improved performance, while a pre-trained ResNet-18 was not

AE	KNN	Pre-trained ResNet-18	Isotope ratio	Relative isotope ratio
×	×	×	0.68	6.50
*✓*	×	×	0.87	7.99
*✓*	*✓*	×	0.90	8.67
*✓*	*✓*	*✓*	0.82	5.76

×/*✓* indicate inclusion/exclusion of each aspect of the training.

We also performed an ablation study on the configurations of the autoencoder. Particularly, our experiments on various dimensions of $z_{i}$ of the autoencoder showed that a smaller dimension of $z_{i}$ may not be able to learn features sufficient to distinguish different clusters. In comparison, a larger dimension of $z_{i}$ captured noises and did not improve the clustering performance significantly (see Supplementary Section S4.3).

### 5.5 Using clusters as inputs to spatial segmentation improved the interpretation in the spatial domain

We investigated the impact of using clusters of *m*/*z* images as input to spatial segmentation. Ion images assigned to a same cluster were segmented with Spatial Shrunken Centroid segmentation (while setting the regularization parameter to 0, to ensure that the segmentation does indeed include all these images as the input).

**The simulated dataset.** Considering the simulated clusters 1 and 2 in Section 4.2, we set out to segment the gray area in Supplementary Figure S1a. The *m*/*z* images in cluster 1 had lower intensity in the big circle, and the *m*/*z* images in cluster 2 had lower intensity in the small circle. When segmenting the images from cluster 2, the segmentation accuracy increased with the increase of the input number of ion images from that cluster. The segmentation accuracy reached its maximum when the input contained the maximum number of ion images from cluster 2, and no images from the other clusters. Further adding input images, and including more ion images from the irrelevant cluster 1 became counterproductive, as the segmentation became dominated by the spatial structure in cluster 1.

**Fig. 5.**
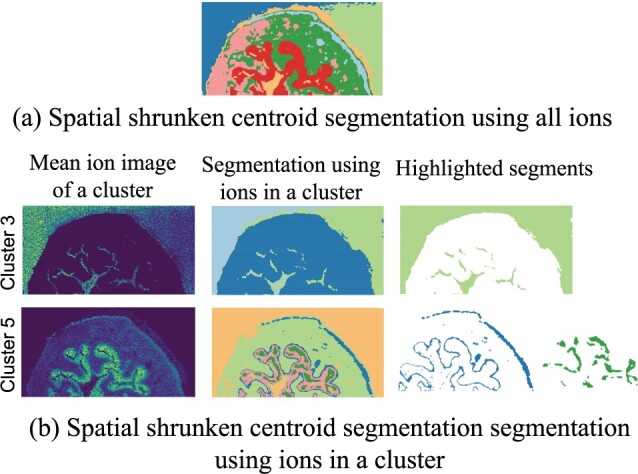
On the mouse bladder dataset, segmentation using ions in a cluster reveals spatial structure. This plot compares the Spatial Shrunken Centroids segmentation of the mouse bladder datasets using all ions (**a**) and only using ions in clusters 3 and 5 of the proposed method (**b**). The highlighted segments were not found when jointly segmenting all the ions

**Mouse urinary bladder dataset.** Figure 5 contrasts the segmentation using all the ions, and using ions from two clusters showing heterogeneous spatial patterns. The mean ion images show the representative spatial distributions of ions in these clusters. While one cluster was enriched in the background, the other was enriched in the urothelium most. The highlighted segments of the first cluster combined segments in red and cyan of segmentation in the middle column. They corresponded to the matrix, which was not intact in the segmentation using all ions. In addition, the segments in red and in purple of the segmentation using the second cluster were corresponding to the basal layer and the umbrella cells, respectively. Both structures were not revealed when the tissue was segmented using all the ions.

**Fig. 6.**
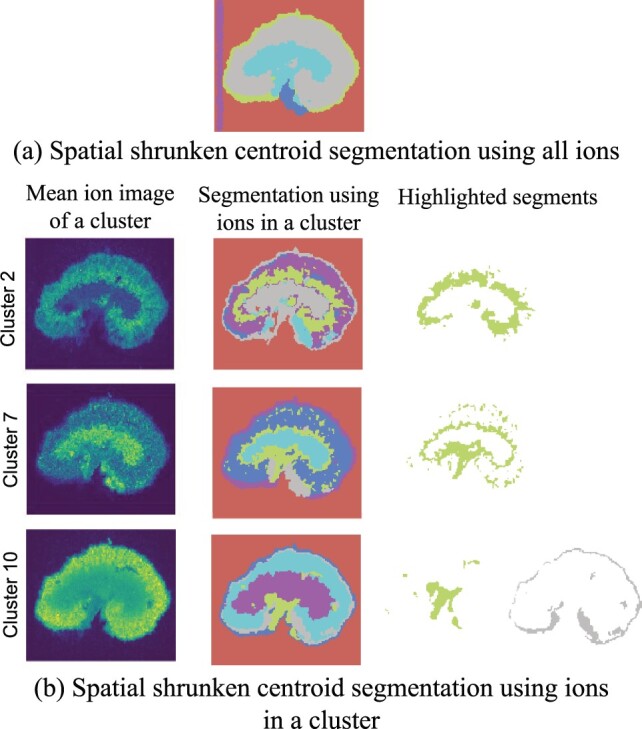
On the mouse kidney dataset, segmentation using ions in a cluster reveals spatial structure. This plot compares the Spatial Shrunken Centroids segmentation of the mouse kidney datasets using all ions (**a**) and only using ions in clusters 2, 7 and 10 of the proposed method (**b**). The highlighted segments were not found when jointly segmenting all the ions

**Mouse kidney dataset.** Figure 6 makes a similar point for the mouse kidney dataset. Segmentation based on clusters of *m*/*z* detected spatial structures that were absent from the segmentation obtained with all the ions. Specifically, the renal pelvis area (the segment in yellow-green for the first and second clusters) and the renal cortex (the segment in yellow-green for the third cluster) were not shown in the all-ions segmentation (Fig. 6a). In addition, the membrane of the kidney presented a layering structure in the segmentation in the bottom row, as it consisted of two segments, the outer segment (blue) and the inner segment (grey).

## 6 Discussion

We proposed a noise-robust deep clustering approach for mass spectrometry images. This approach exploits a convolutional autoencoder to denoise ion images. Pairwise pseudo-labels are assigned to ion image pairs as indicators of whether belonging to a same cluster. They are then used to construct the loss function, which is minimized using an adaptive self-paced learning strategy. This allows the model to gradually learn the clusters from easy to ambiguous examples, thus more effective. This approach is also easy to scale to different image sizes and many ions.

The proposed approach improved the interpretability of *m*/*z* from a same source in the spectral domain as compared to the existing methods on the simulated dataset and four MSI experimental datasets. The improvement came from training the networks on the ion images directly, and from denoising the images prior to learning the features. According to the ablation study of the kidney dataset, a pre-trained ResNet-18 did not improve the clustering performance. This may be due to the fact that MSI images are very different from natural images.

In addition, we qualitatively demonstrated that using ions in a cluster as input to the SSC segmentation improved the interpretability of data in the spatial domain. Spatial segmentation using ions in a cluster revealed spatial structures that were not detected in the all-ion segmentation. The structures were meaningful and could be related to anatomic structures. The results indicated that selecting the right number and type of ions helps improve biological insight. A limitation of the proposed approach is the need to pre-specify the number of clusters, which may require some prior knowledge. This aspect can be improved in future work. Nevertheless, even in its current form the proposed approach can greatly improve the interpretability of MSI data in both spectral and spatial domains.

## Supplementary Material

btad067_Supplementary_Data
