# Supplementary material for: A noise-robust deep clustering of biomolecular ions improves interpretability of mass spectrometric images
Source: Bioinformatics. 2023 Feb 6;39(2):btad067. doi: 10.1093/bioinformatics/btad067 (PMC9942547; doi:10.1093/bioinformatics/btad067)
Supplement: btad067_Supplementary_Data [file btad067_supplementary_data.pdf]

S1 Background

S1.1 Preprocessing data from MSI experiments

Since the experimental variability from sample preparation and data acquisition can introduce chemical noise, intensity variations, and mass shifts [Ràfols et al., 2018], it is necessary to pre-process raw MSI signals prior to clustering or segmentation. Preprocessing such as baseline reduction, peak picking, peak alignment, peak filtering, and binning help remove the artifacts. It can also impact the downstream data interpretations. In the following, we assume that the MSI data are appropriately preprocessed.

S1.2 Unsupervised clustering of the spectral domain

Early methods for ion clustering viewed ion images as vectors, without considering the relative spatial localization of the corresponding pixels. For example, Alexandrov *et al.* [Alexandrov et al., 2013] scaled the ion images such that the sum of the intensities over all the pixels equals 1, represented the scaled values of all the pixels in a vector, and clustered the vectors with a Gaussian Mixture Model (GMM). Wüllems *et al* [Wüllems et al., 2019] defined the similarity score of two m/z in terms of Pearson correlation of their image vectors, and grouped m/z with similar correlations. Unfortunately, treating ion images as vectors does not adequately represent the spatial morphological structure of the tissue.

More recently, inspired by the ability of neural networks to learn complex spatial patterns, Zhang *et al* [Zhang et al., 2021] exploited the Xception [Chollet, 2017] network pre-trained on ImageNet [Deng et al., 2009] to extract high-level ion image features. The authors referred to these features as “neural ion image". The features were used as input to Uniform Manifold Approximation and Projection (UMAP), Density-Based Spatial Clustering of Applications with Noise (DBSCAN), and K-means, and demonstrated better clustering than when clustering the original ion images.

S2 Data

S2.1 Simulated datasets

Mathematically, ion image i ($i = 1, ..., 700$) was simulated as: $x_i^j = \mu_{mi} + \phi_i^j + \epsilon_i^j$, where x_i^j is the intensity of pixel j ($j = 1, ..., 1600$), and μ_{mi} is the mean intensity of segment m ($m \in (1, 2, 3)$) in ion image i. The term ϕ^j expressed the spatial auto-correlation $\phi^j \sim ICAR(\tau^2, W)$ [Bemis et al., 2019], with standard deviation $\tau = 5$. W is the $J \times J$ matrix that takes values of 1 for pairs of locations that are direct neighbors, and 0 otherwise. J is the total number of pixels. ϵ^j is the random noise of pixel j, $\epsilon^j \sim N(0, \sigma_\epsilon^2)$, $\sigma_\epsilon = 0.15\mu_{mi}$.

S2.2 Experimental datasets

Mouse urinary bladder The dataset was acquired using MALDI ionization source at 10 μm spatial resolution and LTQ orbitrap mass spectrometer on positive mode. The raw dataset consisted of 134 $\times$ 260 pixels and 91,630 m/z ranging from 400 Da to 1,000 Da. The preprocessing produced 746 m/z features.

Mouse kidney The dataset was acquired using MALDI ionization source and orbitrap on positive mode. It contained 125 $\times$ 110 mass spectra, and each mass spectrum had 155,400 m/z features ranging from 100 Da to 1210 Da. After preprocessing, it had 4,123 m/z features.

Mouse brain The dataset was acquired using MALDI ionization source and orbitrap as the mass analyzer in negative mode. This dataset was light in the spectra domain (only 1772 m/z features), but rich in the spatial domain (22,302 pixels). The preprocessing produced 350 m/z features.

Rhesus macaque lymph node The dataset was acquired using IR-MALDESI ionization source and Orbitrap as the mass analyzer in positive mode. The raw data contained 703,766 m/z features and 8,704 pixels. The preprocessing produced 15,677 m/z features. The 1000 highest abundant m/z features were kept for the evaluation.

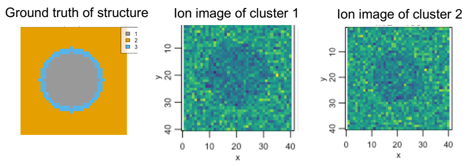

(a) Simulated spatial structure

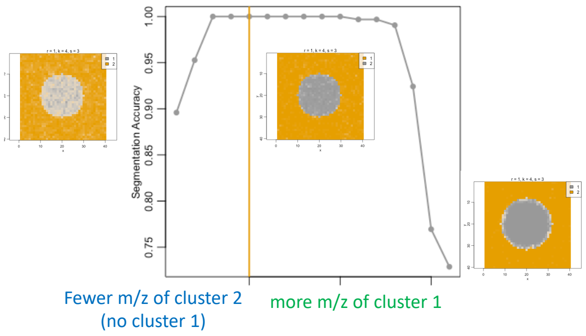

(b) Segmentation accuracy for different groups of ions

Fig. S1: **On the simulated dataset, interfering ions undermined the accuracy of spatial segmentation.** (a) The simulated structure and representative ion images. (b) The plot of segmentation accuracy along with the number of interfering ions. When adding more ions of cluster 1, the segmentation of the gray area in (a) becomes less accurate.

S3 Evaluation strategy

S3.1 Implementation of compared methods

For methods do not account for spatial contextual features of ion images, we re-implemented K-means and GMM with diagonal covariance (Alexandrov *et al.* [Alexandrov et al., 2013]) using Python library *sklearn*.

We further compared the proposed approach to representation-based methods that account for the spatial features of the images. To this end, we re-implemented a Variational Autoencoder (VAE) using PyTorch. The embedding dimension of VAE was set to 10. It was used as a feature extractor, and was followed by K-means clustering. We also re-implemented the method of using Xception network (Zhang *et al.* in [Zhang et al., 2021]) as feature extractor, followed by K-means clustering. To this end, we downloaded from PyTorch image models using the library *timm* the Xception network pre-trained on ImageNet. The output of the last layer before the fully connected layer with a dimension of 2048 was used as the feature representation for ion image clustering.

S3.2 Definition of clustering accuracy (ACC)

Since the predicted cluster label may be different from the ground truth label of a same cluster, ACC is defined as the maximum accuracy for any mapping $m(\cdot)$ between the predicted clustering labels and the ground truth labels. Let c_i denotes the predicted label of ion image i and c_i^{gt} denotes

the ground truth label. ACC is defined as

$$ACC = max_m \frac{1}{n}\sum_{i=1}^{n} \mathbb{1}_{\{m(c_i)=c_i^{gt}\}} \quad (1)$$

Higher ACC indicates more accurate clustering of m/z from a same source.

S4 Additional results

S4.1 Using clusters as inputs to spatial segmentation improved the interpretation in the spatial domain

The simulation example is shown in Figure S1. It shows that adding more interfering ions as input decreased the segmentation accuracy.

S4.2 Ion image clustering helped distinguish tissue-specific and background-specific m/z

It is important to exclude from the downstream analyses ions that are only associated with the background. This reduces the noise and the dimensionality of the data and lowers the computational cost. Unfortunately, selecting tissue-specific ions based on ion intensity might not work very well. Figure S2(a) shows the mean spectrum of the mouse bladder dataset, with shapes indicating cluster labels. A high-intensity m/z was as likely to be associated with the background as with the tissue area. In particular, the two m/z from cluster 6 in the upper box in Figure S2(a) concentrated in the background, while the two m/z from cluster 2 in the lower box concentrated in the muscle area of interest. Figure S2(b) plots the distribution of clustering membership in the mean spectrum of the mouse kidney dataset. It also shows that the m/z of the highest intensity can be both background-specific and tissue-specific ions. The results indicated that selecting tissue-specific m/z based solely on feature intensity is insufficient. On the other hand, clustering groups together m/z with spatial distributions that are more indicative of the tissue (such as cluster 2 in Figure 8(a) or cluster 10 in Figure 8(b)) versus the background (such as cluster 6 in Figure 8(a) or cluster 8 in Figure 8(b)). Therefore, the results of ion image clustering can be exploited as a feature selection method to filter out the background ions, and to select ions specific to a particular tissue region.

S4.3 Ablation study on the dimension of $\mathbf{z}_i$ of the autoencoder

We performed an ablation study on various dimensions of the z layer of the convolution autoencoder. The clustering results on the simulated dataset are shown in Table S1. With the dimension of the z layer as 2, the autoencoder was not able to learn features sufficient to distinguish different clusters except for cluster 3 and cluster 4 (see Figure S4); thus, the clustering CNN achieved a lower clustering accuracy of 0.687. However, the clustering accuracy was not improved but slightly decreased by further increasing the dimension of the z layer from 30 to 256. From the t-SNE plot shown in Figure S4, the learned features of the z layer did not further separate different clusters by increasing the dimension of the z layer.

	dim = 2	dim = 7	dim = 30	dim = 100	dim = 256
ACC	0.687	0.950	0.907	0.923	0.867
NMI	0.693	0.890	0.886	0.878	0.859
ARI	0.560	0.850	0.826	0.835	0.800

Table 1. Clustering performance for different dimensions of $\mathbf{z}_i$ of the autoencoder.

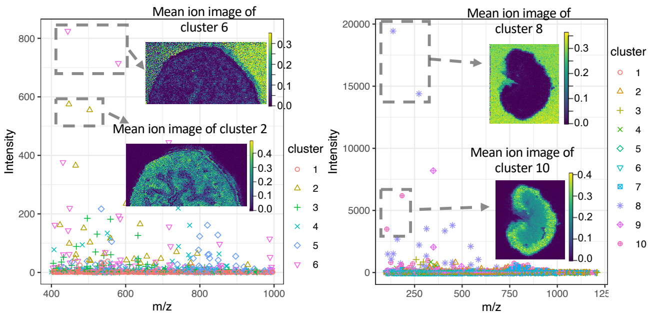

(a) The mouse bladder dataset (b) The mouse kidney dataset

Fig. S2: **On the mouse bladder and kidney datasets, ion image clustering helps select features associated with tissues.** X-axis: m/z. Y-axis: mean intensity across the whole tissue. The colors and shapes of the points in the scatter plot are clustering labels of each m/z. Inset: mean ion images of clusters in which darker colors correspond to lower intensities. The ion images in the inset were normalized to [0,1], such that the mean ion images are less affected by high-intensity artifacts. (a) Mouse bladder dataset. High-intensity ions in cluster 6 may not be of interest for downstream analysis since they are specific to the background. (b) As (a), mouse kidney dataset. Similarly, high-intensity ions in cluster 8 may not be of interest for downstream analysis since they are specific to the background.

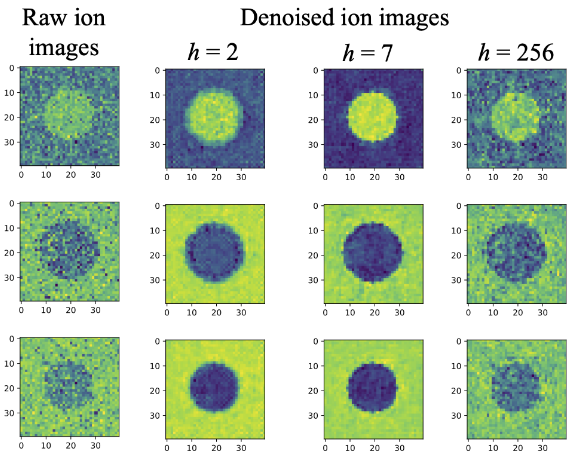

Fig. S3: The denoised ion images for different dimension of the z layer of the autoencoder. h denotes the dimension of $\mathbf{z}_i$. With a small h, the autoencoder did not reconstruct the spatial structure of the ion image shown in row 1. However, with a large h, the reconstructed images shown in column 3 contain significantly larger noises than a smaller h.

	Experiment 1	Experiment 2	Experiment 3	Experiment 4
ACC	0.954	0.927	0.957	0.979
NMI	0.928	0.891	0.917	0.959
ARI	0.902	0.854	0.904	0.952

Table 2. For four repeated experiments with the simulated dataset, the average clustering accuracy was 0.954 and the standard deviation was 0.021.

S4.4 Variations of model predictions

The weight initializations of the neural networks follow a random distribution, and the backpropagation of gradients also contains randomness. As a result, the trained model has random variations in model weights, affecting model performance and predictions. We repeated

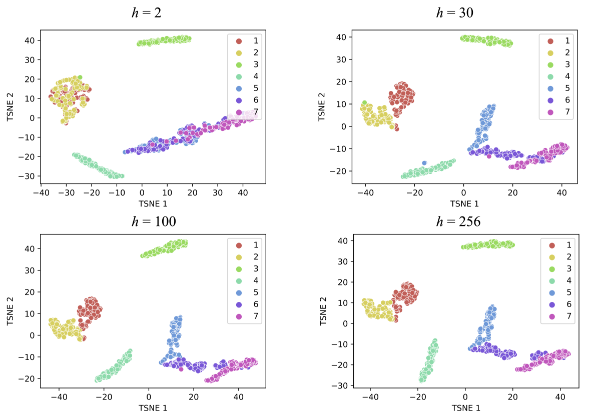

Fig. S4: The t-SNE plot of the z layer outputs of the convolutional autoencoder. h is the dimension of the z layer. When h , the encoder is not able to learn the embeddings of images that are sufficient to distinguish different clusters except for cluster 3 and cluster 4. However, increasing h from 30 to 256 does not further help separate different clusters.

	Experiment 1	Experiment 2	Experiment 3	Experiment 4
Cluster 1	4	3	3	5
Cluster 3	0	1	12	0
Cluster 6	22	35	9	10

Table 3. The number of mis-clustered images in three clusters for four repeated experiments. The cluster membership 1, 3, and 6 refer to the ground truth of the simulated dataset shown in Figure 3 in the main text.

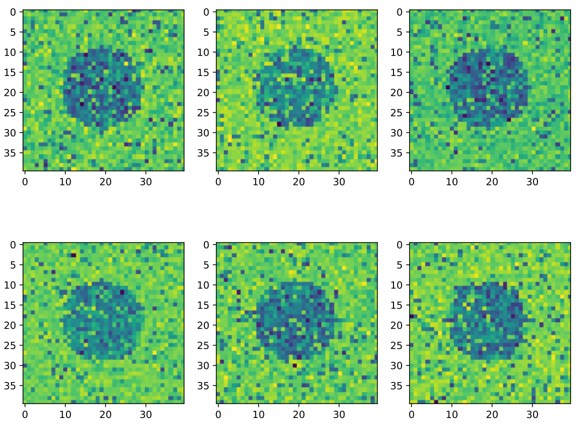

Fig. S5: The ion images in cluster 6 that are consistently mis-clustered for four repeated experiments.

the training on the simulated dataset four times and show the results in Table 2. The average clustering accuracy was 0.954, and the standard deviation was 0.021. The detailed number of mis-clustered images for the repeated experiments are shown in Table 3. Images from cluster 4 are of homogeneous distribution and easier to cluster; thus, they are correctly clustered by different runs consistently. Images from cluster 6 are more difficult to cluster as they are very similar to images from clusters 5 and 7. Figure 4 shows the ion images in cluster 6 that are consistently mis-clustered in four repeated runs. They are Representative of difficult samples.

References

T. Alexandrov, I. Chernyavsky, M. Becker, F. von Eggeling, and S. Nikolenko. Analysis and interpretation of imaging mass spectrometry data by clustering mass-to-charge images according to their spatial similarity. *Analytical Chemistry*, 85:11189, 2013.

K. A. Bemis, D. Guo, A. J. Harry, M. Thomas, I. Lanekoff, M. P. Stenzel-Poore, S. L. Stevens, J. Laskin, and O. Vitek. Statistical detection of differentially abundant ions in mass spectrometry-based imaging experiments with complex designs. *International Journal of Mass Spectrometry*, 437:49–57, 2019.

F. Chollet. Xception: Deep learning with depthwise separable convolutions. In *Proceedings of the IEEE conference on computer vision and pattern recognition*, pages 1251–1258, 2017.

J. Deng, W. Dong, R. Socher, L. Li, K. Li, and F. Li. Imagenet: A large-scale hierarchical image database. In *2009 IEEE conference on computer vision and pattern recognition*, 2009.

P. Ràfols, D. Vilalta, J. Brezmes, N. Cañellas, E. Del Castillo, O. Yanes, N. Ramírez, and X. Correig. Signal preprocessing, multivariate analysis and software tools for ma (ldi)-tof mass spectrometry imaging for biological applications. *Mass spectrometry reviews*, 37(3):281–306, 2018.

K. Wüllems, J. Kölling, H. Bednarz, K. Niehaus, V. H. Hans, and T. W. Nattkemper. Detection and visualization of communities in mass spectrometry imaging data. *BMC Bioinformatics*, 20:1, 2019.

W. Zhang, M. Claesen, T. Moerman, M. R. Groseclose, E. Waelkens, B. De Moor, and N. Verbeeck. Spatially aware clustering of ion images in mass spectrometry imaging data using deep learning. *Analytical and Bioanalytical Chemistry*, 413:2803, 2021.
